# Cell death and restoration of TRAIL-sensitivity by ciglitazone in resistant cervical cancer cells

**DOI:** 10.18632/oncotarget.22632

**Published:** 2017-11-22

**Authors:** Marie-Laure Plissonnier, Sylvie Fauconnet, Hugues Bittard, Christiane Mougin, Jean Rommelaere, Isabelle Lascombe

**Affiliations:** ^1^ EA3181, Université Bourgogne Franche-Comté, LabEx LipSTIC ANR-11-LABX-0021, Besançon F-25030, France; ^2^ Department of Urology, University Hospital of Besançon, Besançon F-25030, France; ^3^ Department of Pathology, University Hospital of Besançon, Besançon F-25030, France; ^4^ German Cancer Research Center Tumor Virology F010, Heidelberg 69120, Germany; ^5^ Cancer Research Center of Lyon, INSERM U1052, Lyon F-69424, France

**Keywords:** cervical cancer cells, apoptosis, PPAR, thiazolidinedione, TRAIL, HPV

## Abstract

Known activators of the Peroxisome Proliferator-Activated Receptor γ (PPARγ), thiazolidinediones (TZD) induce apoptosis in a variety of cancer cells through dependent and/or independent mechanisms of the receptor. We tested a panel of TZD (Rosiglitazone, Pioglitazone, Ciglitazone) to shed light on their potential therapeutic effects on three cervical cancer cell lines (HeLa, Ca Ski, C-33 A). In these cells, only ciglitazone triggered apoptosis through PPARγ-independent mechanisms and in particular *via* both extrinsic and intrinsic pathways in Ca Ski cells containing Human PapillomaVirus (HPV) type 16. It also inhibits cervical cancer xenograft development in nude mice. Ciglitazone kills cervical cancer cells by activating death receptor signalling pathway, caspase cascade and BH3 interacting-domain death agonist (Bid) cleavage through the up-regulation of Death Receptor 4 (DR4)/DR5 and soluble and membrane-bound TNF related apoptosis inducing ligand (TRAIL). Importantly, the drug let TRAIL-resistant Ca Ski cells to respond to TRAIL through the downregulation of cellular FLICE-Like Inhibitory Protein (c-FLIP) level. For the first time, we revealed that ciglitazone is able to decrease E6 viral oncoprotein expression known to block TRAIL pathway and this was associated with cell death. Our results highlight the capacity of ciglitazone to restore TRAIL sensitivity and to prevent E6 blocking action to induce apoptosis in cervical cancer cells.

## INTRODUCTION

Cervical cancer is the third most commonly diagnosed cancer and the fourth leading cause of cancer death in women worldwide [1]. Persistent infection of high risk human papillomaviruses (HPV) such as HPV16 and 18 is responsible for more than 70% of all cases [2]. Two virally encoded oncoproteins E6 and E7 contribute to carcinogenesis by decreasing pivotal cell signalling components, including the tumour suppressors p53 and pRb that lead to the dysregulation of cell proliferation, apoptosis and genome instability [3]. HPV E6 protein binds to p53 and favours its degradation by the proteasome through the formation of a complex with E6-associated protein (E6AP), a member of the E3-ubiquitin ligase family. HPV E7 protein interacts with pRb, inducing its proteolytic degradation leading to the destabilization and the disruption of Rb/E2F repressor complexes which results in increased transcription of E2F-responsive genes and S-phase cell cycle progression [4]. The development of therapeutic approaches which can specifically restore the cellular death pathways inactivated by the viral oncoproteins, appears to be fundamental for the clinical treatment of HPV-induced cervical cancer.

TRAIL (TNFα-related apoptosis inducing ligand) is a hopeful anti-neoplastic agent because it induces cancer cell death without toxicity on normal cells [5]. TRAIL initiates apoptotic process through interaction with the death receptors 4 and 5 (DR4 and DR5), leading to the formation of the death-inducing signalling complex (DISC), the recruitment and rapid activation of caspase 8. Cleaved caspase 8 induces cleavage of Bid (BH3 interacting-domain death agonist), followed by mitochondrial-dependent activation of caspase 9 through the release of cytochome c [6] that cooperates with apoptotic protease-activating factor-1 (Apaf-1). Both caspases 8 and 9 activate the executioner caspase 3, which is the primary activator of apoptotic DNA fragmentation and leads to cancer cell apoptosis [7]. Despite the numerous reports describing the favourable anti-tumour activities of TRAIL, some cancer cells are refractory to TRAIL-induced apoptosis. Therapeutic approaches based on the combination of TRAIL with different chemotherapeutic agents have been developed to overcome the resistance of tumour cells to TRAIL [8].

Thiazolidinediones (TZD), including rosiglitazone (Avandia^®^), troglitazone (Rezulin^®^), pioglitazone (Actos^®^) and ciglitazone are antidiabetic agents developed to treat type II diabetes [9, 10]. They are activators of Peroxisome Proliferator-Activated Receptor γ (PPARγ) [11] which is a ligand-activated transcription factor belonging to the nuclear receptor superfamily. In response to various stimuli (fatty acids and their metabolites, xenobiotics), PPAR-regulated transcription involves the heterodimerization with retinoid X receptors (RXR) and the binding to a peroxisome proliferator responsive element (PPRE) located in the promoter region of target genes [12]. TZD-mediated activation of PPARγ regulates glucose homeostasis and lipid metabolism, promotes adipocyte differentiation and controls inflammation [13–15]. In addition, TZD exert antitumour activities in different cancer cell lines (prostate, thyroid, colon, breast, lung...), animal models, and clinical trials [16]. The anticancer effects of TZD are associated with their ability to suppress cell growth and invasion, and to promote apoptosis [17]. Interestingly, TZD could overcome the TRAIL resistance of tumour cells. But, the underlying mechanisms remain incompletely understood. No data on TZD-mediated TRAIL sensitivity restoration are available in cervical cancer cells.

In this study, we investigated the pro-apoptotic activity of TZD in several cervical cancer cell lines (HeLa, Ca Ski, C-33 A) and demonstrated that only ciglitazone individual treatment induced Ca Ski cell apoptosis through PPARγ-independent mechanisms. Importantly, we showed that this PPARγ agonist reduced xenografted tumour growth in nude mice. Interestingly and for the first time in Ca Ski cells, we revealed that ciglitazone induced death through the upregulation of TRAIL and DR4/DR5 whereas these cells were resistant to TRAIL. We displayed that the combination of ciglitazone and TRAIL allowed TRAIL-refractory Ca Ski cells to respond to TRAIL through a marked decrease of c-FLIP due to a proteasomal degradation process and an inhibition of translation initiation. In addition, ciglitazone downregulated HPV16 E6 at the mRNA and protein level.

## RESULTS

### Ciglitazone induces cervical cancer cell death

**Figure 1:**
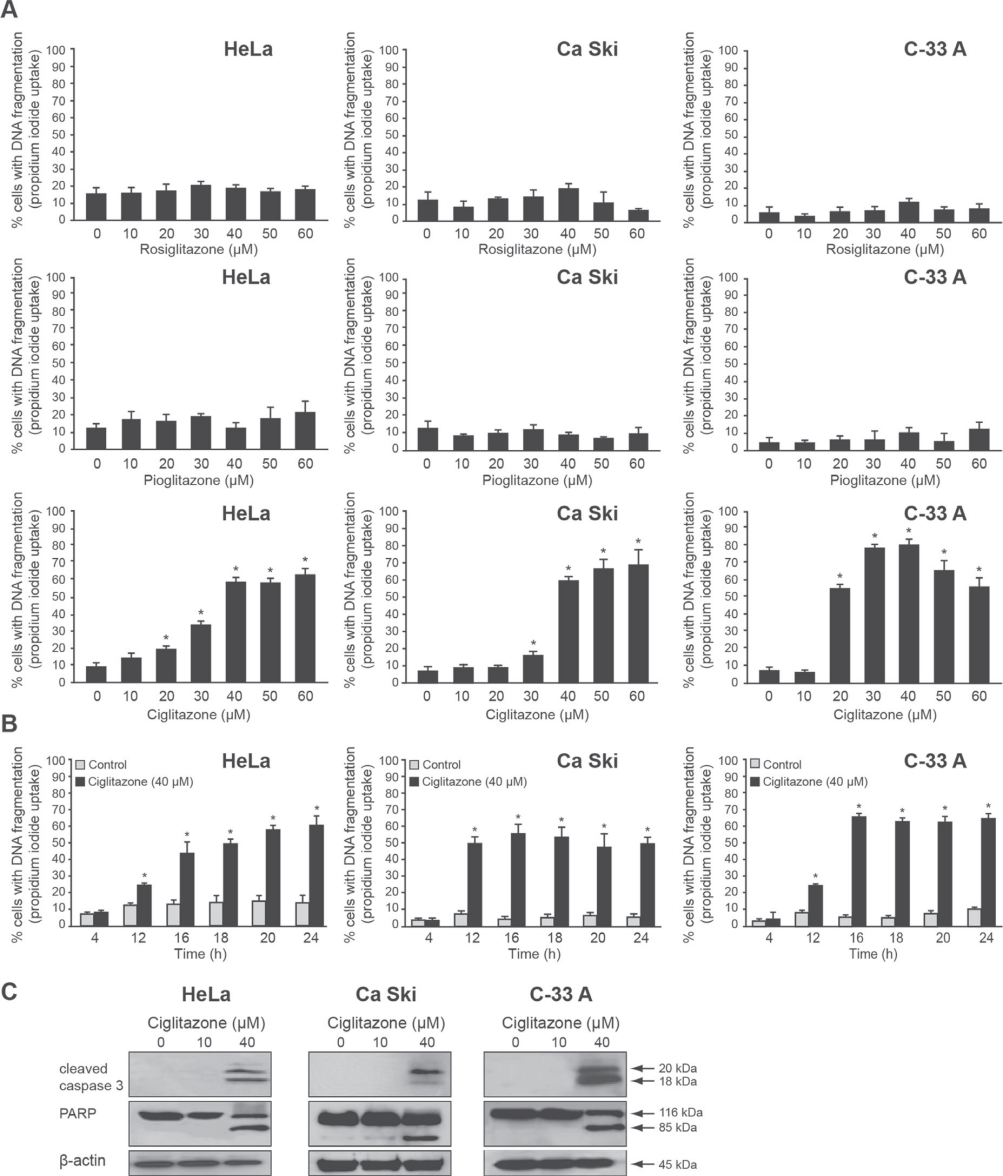
Ciglitazone induces human cervical cancer cell apoptosis (**A**) Dose-dependent effect of TZD (rosiglitazone, pioglitazone, ciglitazone). HeLa, Ca Ski and C-33 A cells were exposed for 24 h to vehicle or TZD at the indicated concentrations. (**B**) Time-dependent effect of ciglitazone. Cells were exposed to 40 μM ciglitazone for the indicated times. (A, B) The percentage of cells showing the hypodiploid DNA content (sub-G1 peak) was evaluated by flow cytometry analysis after propidium iodide staining. (**C**) The cleavage of caspase 3 and PARP was determined by western blotting analysis. β-actin was used as an internal loading control. Data are means ± SEM of three independent experiments performed in triplicates. ^*^P < 0.05 compared to control cells.

To determine whether TZD could induce cervical cancer cell death, HeLa (HPV18), Ca Ski (HPV16), and C-33 A (HPV negative) cells were treated with increasing concentrations of rosiglitazone, pioglitazone and ciglitazone for 24 h and assessed for PI stained DNA content using flow cytometry. Only ciglitazone led to an increase of the sub-G1 cell population in a dose-dependent manner from a concentration of 20 μM (HeLa, C-33 A) or 30 μM (Ca Ski) (Figure 1A). At the concentration of 30 μM ciglitazone, we noticed that the percentage of cell death was higher in HPV negative C-33 A cells (80%) compared to HeLa (30%) and Ca Ski (15%) lines which are HPV positive cells. For the concentrations higher than 30 μM, the response variability of the three cell lines to ciglitazone exposure was less marked. As shown in Figure 1B, 40 μM ciglitazone increased the percentage of cells with fragmented DNA from 12 h exposure for the three cell lines. At 24 h, 60% of HeLa cells, 50% of Ca Ski cells and 65% of C-33 A cells with DNA fragmentation were observed. As determined by western blotting, caspase 3 was cleaved upon 40 μM ciglitazone treatment and its activation was confirmed by the cleavage of its downstream substrate PARP (Figure 1C).

### Ciglitazone acts through PPARγ-independent mechanisms

**Figure 2:**
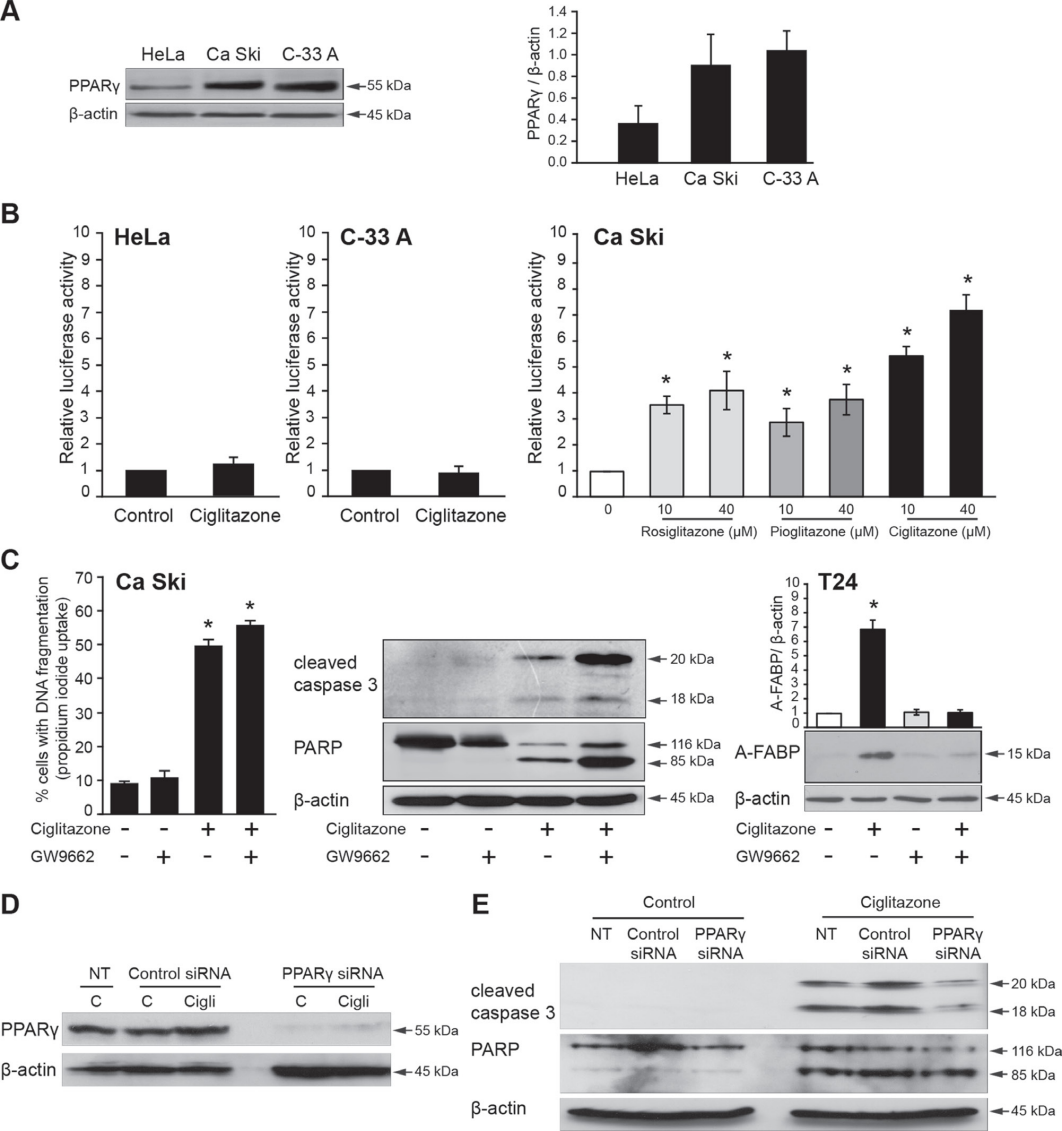
PPARγ-independent effects of ciglitazone in Ca Ski cells (**A**) Western blot analysis of PPARγ expression in HeLa, Ca Ski and C-33 A cervical cancer cell lines. (**B**) Luciferase activity in cells cotransfected with Cyp2XPal-luc firefly and *Renilla* luciferase reporter genes as described in Materials and methods and treated for 12 h with 40 μM ciglitazone (HeLa, C-33 A), 10 or 40 μM rosiglitazone, pioglitazone or ciglitazone (Ca Ski). (**C**) Ca Ski cells were treated for 12 h by 40 μM ciglitazone alone or in combination with 80 μM GW9662, an irreversible potent inhibitor of PPARγ. *Left*, the percentage of cells showing hypodiploid DNA content (sub-G1 peak) was evaluated by flow cytometry analysis; *middle*, after treatment, whole cell lysates were prepared and total protein extracts were subjected to immunoblotting for detection of procaspase 3 and PARP processing; *right*, positive control : GW9662 blocking action on the ciglitazone-mediated expression of the A-FABP (PPARγ target gene). β-actin was used as an internal loading control. (**D**, **E**) Effect of PPARγ knockdown on ciglitazone-induced Ca Ski cell apoptosis. Cells were transfected with PPARγ siRNA or non-targeting siRNA and treated or not with 40 μM ciglitazone. PPARγ expression as well as procaspase 3 and PARP processing were analysed by western blotting from whole cell protein extracts. Data are means ± SEM of three independent experiments performed in triplicates. ^*^P < 0.05 compared to untreated cells.

PPARγ was expressed in the three cell lines but to a higher extent in both Ca Ski and C-33 A cells compared to HeLa cells (Figure 2A). As evidenced by different TZD (rosiglitazone/pioglitazone/ciglitazone)-stimulated expression of a PPRE-driven luciferase construct, the receptor was functional only in Ca Ski cells (Figure 2B). It should be noted that ciglitazone was more effective at the tested concentrations to increase luciferase activity. Thus, the effect of ciglitazone on HeLa and C-33 A cell death was PPARγ-independent since in these cells the receptor was not activated by ciglitazone. To examine whether PPARγ transcriptional activity was needed for ciglitazone-promoted cell death in Ca Ski cell line, cells were stimulated for 12 h by 40 μM drug alone or in combination with 80 μM GW9662, an irreversible potent inhibitor of PPARγ. The impact of ciglitazone on cell death (Figure 2C, left panel), caspase 3 and PARP cleavage (Figure 2C, middle panel) was not blocked by the addition of the PPARγ antagonist. Thus, GW9662 had no inhibitory effect on ciglitazone-mediated cell death; and yet it was efficient since it inhibited overexpression of the A-FABP PPARγ target when it was associated with ciglitazone in T24 bladder cancer cells (Figure 2C, left panel) as already described [18]. We then applied a RNA interference strategy to knockdown PPARγ protein. Vehicle- or ciglitazone-treated cells were transfected with a nonspecific control siRNA or PPARγ siRNA. In our transfection conditions, PPARγ protein level was efficiently inhibited (Figure 2D) upon PPARγ silencing in control cells as well as in the presence of ciglitazone. However, the apoptotic effect of ciglitazone was not suppressed by PPARγ knockdown since caspase 3 and PARP were still cleaved (Figure 2E). Taken together, these results indicate that ciglitazone induces apoptotic cell death through PPARγ-independent mechanisms in cervical cancer cells. In the following experiments we focused our study on the effect of ciglitazone in Ca Ski cells.

### Ciglitazone inhibits Ca Ski xenograft tumour growth in nude mice

**Figure 3:**
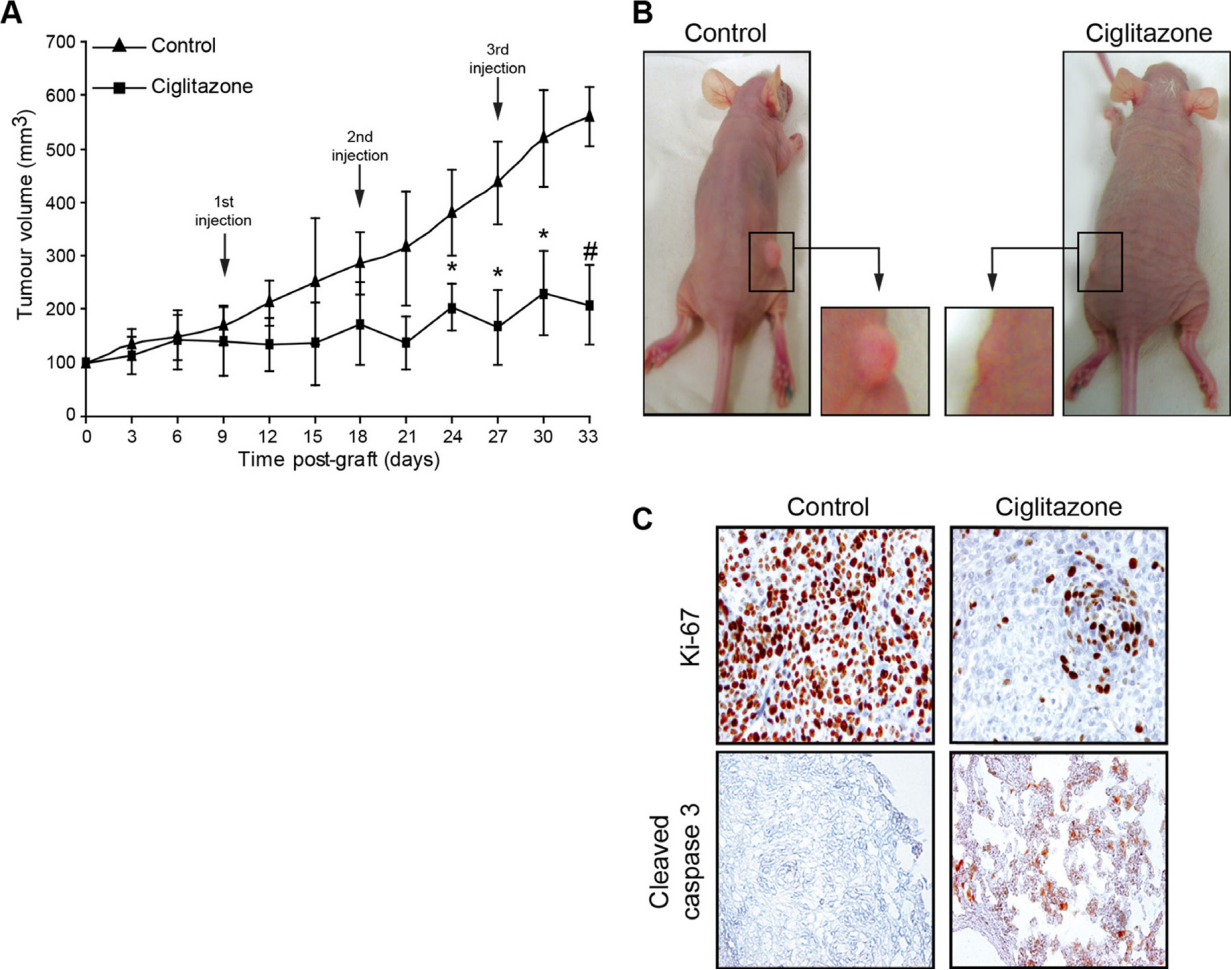
Antitumour activity of ciglitazone administered as monotherapy in athymic mice bearing Ca Ski cervical cancer xenografts Mice were inoculated subcutaneously with exponentially growing Ca Ski cervical cancer cells (5 × 10^6^ cells). When tumour size reached 40 mm^3^ in volume, mice were randomly divided into control and treated groups (*n* = 10). Intraperitoneal injections of ciglitazone were weekly administered at a dose of 15 mg/kg during three weeks. Control animals received only saline vehicle following an identical schedule. (**A**) The growth tumour curve was determined by measuring the tumour volume. ^*^P < 0.05 compared to vehicle-treated animals with the use of two-way ANOVA test (evaluation of the tumour volume development over time). ^#^*P* < 0.05 significant differences between control and treated mice at each post-graft time with the use of two-tailed unpaired Student's *t*-test. (**B**) Photographs showing the decrease of the tumour volume after ciglitazone treatment. (**C**) Immunohistochemical staining of representative paraffin-embedded sections of tumours from untreated or ciglitazone-treated mice. Sections were fixed and stained for Ki-67 and active caspase 3. Each panel is representative of 5 sections for each of ten tumours from control and ciglitazone-treated mice. Original magnification, x20.

To analyse the ciglitazone anticancer effect *in vivo*, Ca Ski cells were subcutaneously injected in 6-week-old-female athymic nude mice. When tumours were palpable, mice received one injection per week of vehicle or ciglitazone (15 mg/kg) for three weeks. Tumour volume was measured twice per week until the sacrifice of mice. The tumour incidence was 100%. Vehicle-treated control animals developed tumours that grew continuously during the treatment period. In contrast, ciglitazone-treated mice exhibited delayed tumour growth development compared to control mice. A significant difference was observed 6 days after the second injection of ciglitazone (Figure 3A, 3B). To investigate whether the inhibition of the tumour growth by ciglitazone was due to inhibition of proliferation, induction of apoptosis, or both, we analysed Ki67 expression and caspase 3 activation by immunohistochemistry (Figure 3C). Ca Ski cell-xenografted tumour sections from ciglitazone-treated mice showed a decrease of Ki67 staining and an activated caspase 3 staining compared to sections of tumours from untreated animals. These results indicate that the decrease of tumour volume observed in ciglitazone-treated animals is related to the inhibition of cell proliferation as well as the induction of apoptotic cell death.

### E6 oncoprotein downregulation contributes to ciglitazone-mediated apoptosis

**Figure 4:**
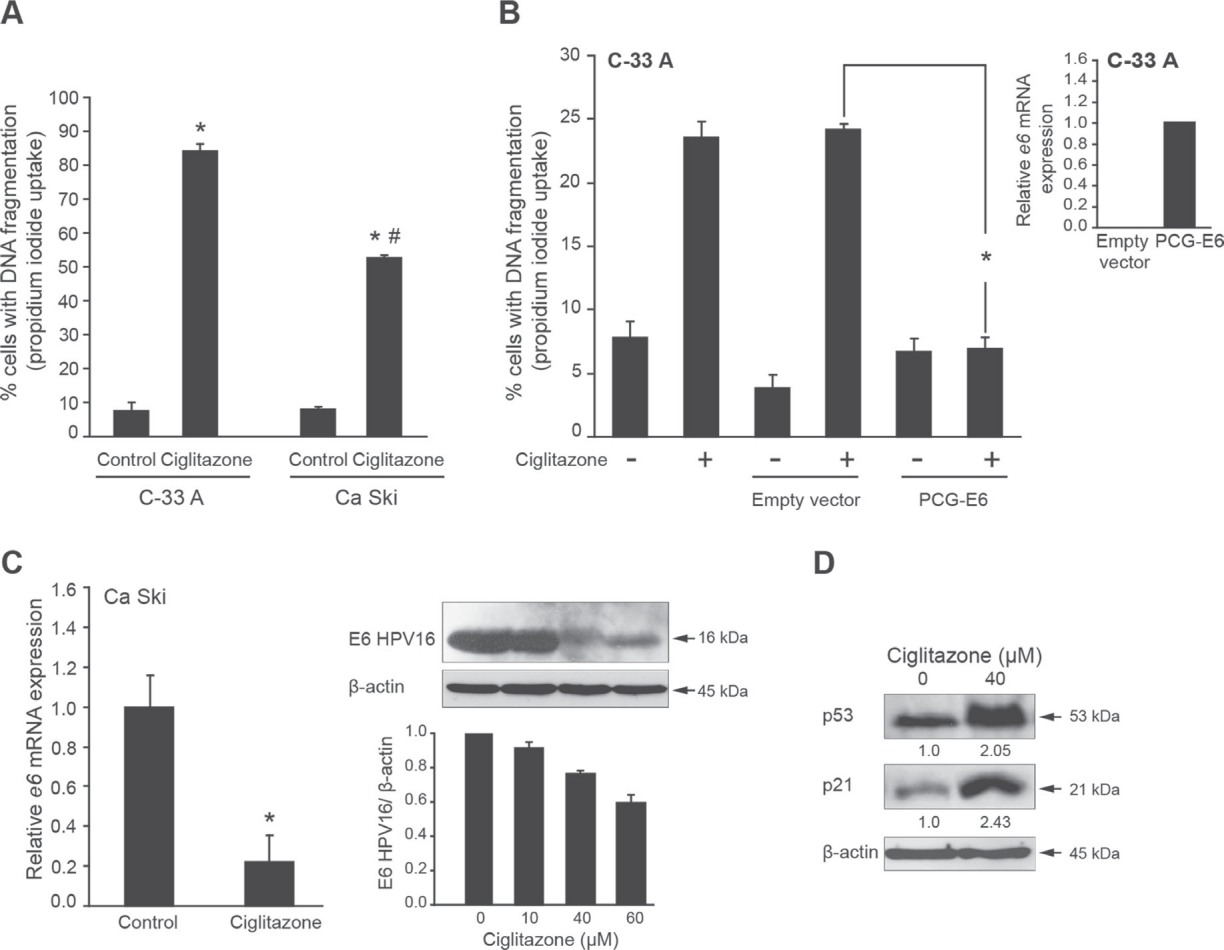
Ciglitazone-mediated Ca Ski cell apoptosis is associated with E6 downregulation (**A**) C-33 A and Ca Ski cells were treated or not with 40 μM ciglitazone for 12 h. The percentage of cells showing hypodiploid DNA content (sub-G1 peak) was evaluated by flow cytometry analysis. ^*^P < 0.05 compared to control cells. ^#^*P* < 0.05 compared to C-33 A cells treated with ciglitazone. (**B**) C-33 A cells were transfected or not with empty vector or with pCG-E6 construct and stimulated with 40 μM ciglitazone for 12 h. The percentage of cells in sub-G1 was evaluated by flow cytometry analysis; *insert*, RTqPCR expression analysis of *e6* transcript levels confirming the efficiency of the pCG-E6 plasmid transfection. ^*^P < 0.05 compared to empty vector-transfected cells treated with ciglitazone. (**C**) *left*, RTqPCR expression analysis of *e6* transcript levels in Ca Ski cells treated or not with 40 μM ciglitazone for 12 h; *right*, western blot of E6 level with β-actin as an internal loading control and densitometric analysis. ^*^P < 0.05 compared to control cells. (**D**) Immunoblotting analysis for p53 and p21 expression. β-actin was used as an internal loading control. Values of densitometric analyses are indicated.

Ca Ski cells are characterized by HPV16 viral infection. E6 oncoprotein is known to block apoptotic process in infected cells [3]. To explore whether E6 is involved in ciglitazone-induced apoptosis, we compared the effect of 40 μM drug on DNA fragmentation in both Ca Ski (HPV positive) and C-33 A (HPV negative) cells (Figure 4A). The percentage of cells showing hypodiploid DNA content (sub-G1 peak) was significantly higher in C-33 A cells than in Ca Ski cells. Thus, we hypothesized that the less sensitivity of Ca Ski cells to ciglitazone-mediated cell death compared to C-33 A cells was due to the presence of E6. To test this hypothesis, C-33 A cells were transfected with pCG-E6 construct encoding E6 viral oncoptotein and treated with ciglitazone (Figure 4B). In Figure 4B (insert), we validated the efficiency of the transfection since we detected *e6* mRNA expression in C-33 A cells. Most importantly, C-33 A cells expressing E6 were resistant to ciglitazone-induced apoptosis compared to untransfected cells as evidenced by a dramatic decrease of cells with fragmented DNA (Figure 4B). To examine the ability of ciglitazone to decrease E6 expression, Ca Ski cells were treated with ciglitazone and E6 level was measured by RTqPCR and western blotting. As presented in Figure 4C (left panel), *e6* transcript level was reduced by up to 80% with 40 μM ciglitazone and the immunoblot analysis confirmed the decrease of E6 protein upon ciglitazone exposure (Figure 4C, middle panel). Since E6 is known to target p53 for proteasomal degradation [4], we thus analysed p53 protein expression level as an indirect control of E6 decrease. In ciglitazone-treated Ca Ski cells, the p53 protein level was increased compared to control cells (Figure 4D, right panel). In addition, we showed that p53 was transcriptionally active by evaluating p21 level, a major transcriptional target of p53. As expected, p21 protein expression was increased after ciglitazone treatment (Figure 4D, right panel). These results indicate that ciglitazone is able to kill Ca Ski cells and this is associated with a decrease of E6 expression. E6 protein could be involved in the less sensitivity of HPV positive cervical cancer cells to ciglitazone.

### Ciglitazone acts through the death receptor and mitochondrial pathways

**Figure 5:**
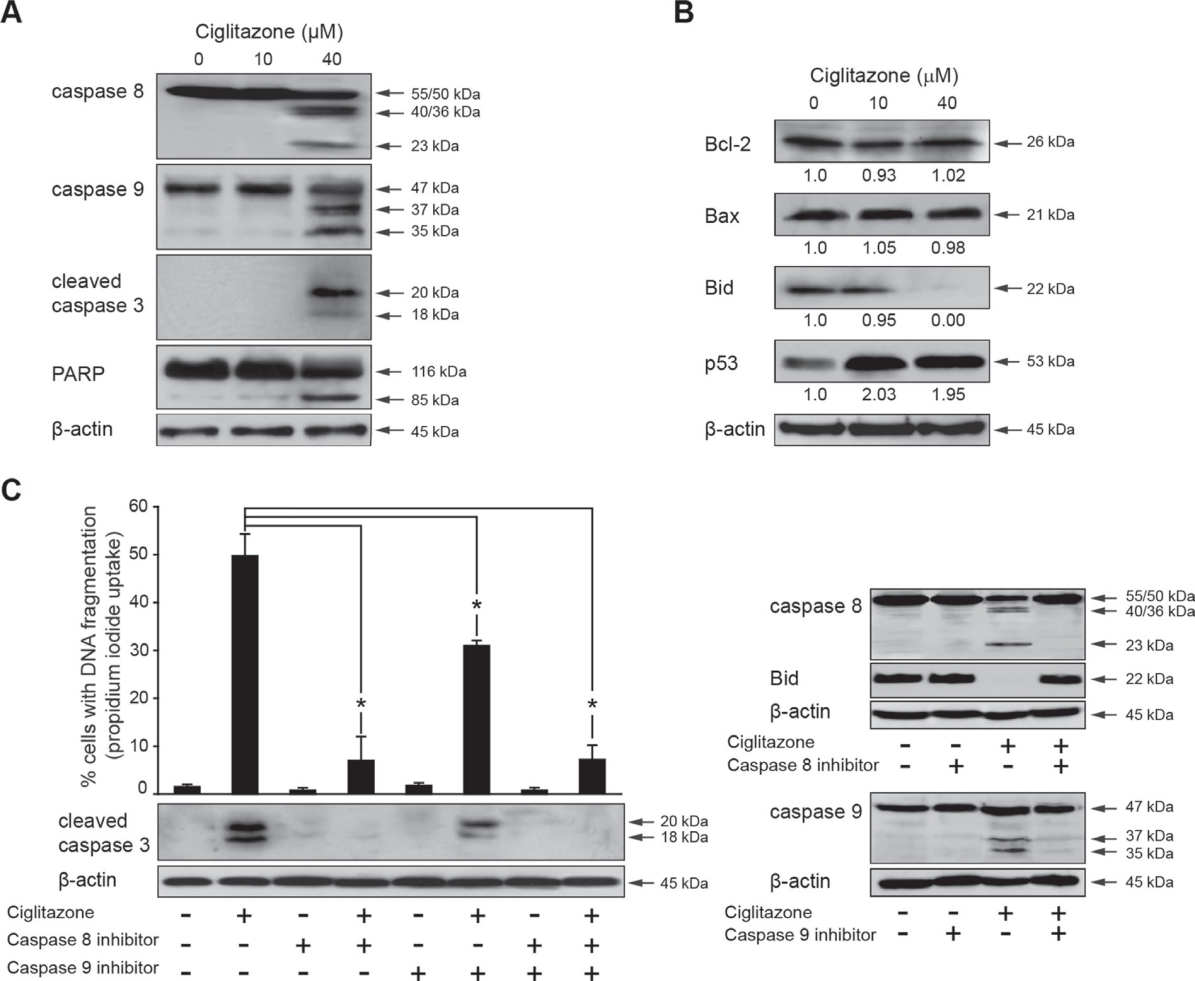
Ciglitazone triggers Ca Ski cell death through extrinsic and intrinsic apoptotic pathways (**A**) Western blotting analysis for the detection of caspase 8, 9, 3 and PARP cleavage after ciglitazone treatment. (**B**) The levels of Bcl-2, Bax, Bid and p53 were assessed by immunoblotting analysis. (**C**) Cells were pre-incubated for 1 h with 50 μM caspase 8 (Z-IETD-FMK) or 9 (Z-LEHD-FMK) specific inhibitor before a 12 h-treatment with 40 μM ciglitazone and stained with PI for DNA fragmentation analysis by flow cytometry. *Bottom and right panels,* Assessment of caspase 3 activation and caspase 8, 9 and Bid processing by western blotting. β-actin was used as an internal loading control. Data are means ± SEM of three independent experiments performed in triplicates. ^*^P < 0.05 compared to ciglitazone-treated cells.

To identify the apoptotic pathway activated by ciglitazone, caspase 8 and 9 cleavage was analysed by western blotting (Figure 5). At a concentration of 40 μM, both caspases were cleaved (Figure 5A), as well as the caspase 3 whose activation was confirmed by the cleavage of PARP (Figure 5A). As shown in Figure 5B, a 2-fold increase in p53 level was observed. In addition, while Bcl-2 and Bax expression levels remained unchanged, Bid was cleaved/activated as revealed by the decrease of the Bid proform. These data revealed that in Ca Ski cells ciglitazone could increase the apoptotic signal through the intrinsic pathway. The specific inhibitors of caspase 8 (Z-IETD-FMK) and caspase 9 (Z-LEHD-FMK) inhibited the ciglitazone-triggered apoptosis as revealed by a significant decrease of cells with fragmented DNA and the inhibition of caspase 3 cleavage (Figure 5C, left panel). It should be noted that in the presence of caspase 8 inhibitor, the caspase 3 was no more cleaved whereas in the presence of caspase 9 inhibitor it was still activated as evidenced by its cleavage indicating that the death receptor pathway was likely predominant. Z-IETD-FMK and Z-LEHD-FMK inhibitors were efficient. Indeed, the caspase 8 cleavage was inhibited in the presence of ciglitazone associated with Z-IETD-FMK and Bid level was restored. In the same way, caspase 9 processing was abolished by the presence of Z-LEHD-FMK in ciglitazone-treated cells (Figure 5C, right panel). These results attest the caspase cascade activation in apoptotic Ca Ski cell death. In addition, the extrinsic apoptotic pathway appears to be dominant for the execution of the ciglitazone-mediated death signal.

### Ciglitazone increases DR4 and DR5 expression at the cell surface

**Figure 6:**
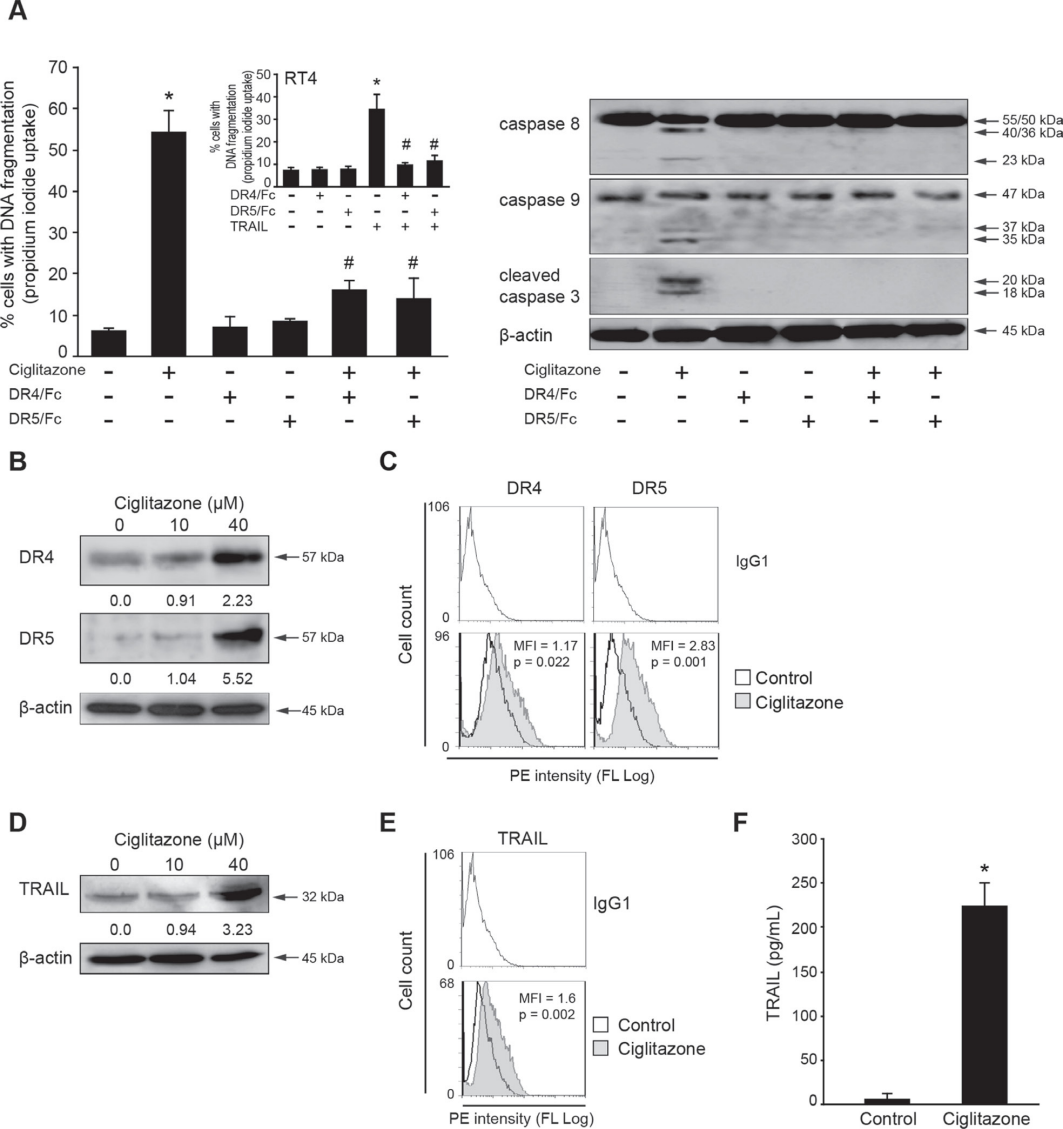
Ciglitazone triggers apoptosis through DR4 and DR5 signalling pathway (**A**) Following a one hour-pre-incubation with or without monoclonal antibodies blocking DR4 and DR5 receptors (5 μg/ml), Ca Ski cells were stimulated by 40 μM ciglitazone and RT4 cells by 50 ng/ml TRAIL (*insert*) for 12 h. The percentage of cells showing hypodiploid DNA content (sub-G1 peak) was evaluated by flow cytometry analysis. Data are means ± SEM of three independent experiments performed in triplicates. ^*^P < 0.05 compared to untreated cells, ^#^*P* < 0.05 compared to ciglitazone-treated Ca Ski cells or TRAIL-treated RT4 cells. *Right*, after treatment, whole cell lysates were prepared and total protein extracts were subjected to immunoblotting for detection of procaspase 8, 9 and 3 processing. (**B**) Cells were treated for 12 h with ciglitazone at 10 and 40 μM. Cellular proteins were isolated and subjected to immunoblotting for detection of DR4 and DR5. Values of densitometric analyses are indicated. (**C**) Ciglitazone-treated cells were stained with anti-DR4-PE or anti-DR5-PE and analysed by flow cytometry. (**D**) Whole cell lysates prepared from ciglitazone-treated cells were assayed for TRAIL expression by western blotting analysis. Values of densitometric analyses are indicated. (**E**) Ca Ski cells were stained with anti-TRAIL-PE and analysed by flow cytometry. (**F**) Conditioned media were collected from ciglitazone-stimulated cells and the concentration of soluble TRAIL was measured by ELISA. Data are means ± SEM of two independent experiments performed in quadruplicates. ^*^P < 0.05 compared to untreated cells. β-actin was used as an internal loading control.

We have highlighted the caspase 8 processing in apoptotic Ca Ski cells. To determine whether the ciglitazone-induced apoptosis was a consequence of death receptor activation, DR4 and DR5 stimulation was inhibited by pre-incubation of cells with blocking anti-DR4 or anti-DR5 before ciglitazone treatment. The inactivation of both receptors significantly inhibited the ciglitazone-promoted apoptosis as evidenced by a significant decrease of the percentage of cells with fragmented DNA (Figure 6A, left panel) and the absence of caspase 8, 9, and 3 processing (Figure 6A, right panel). To confirm the efficiency of the death receptor blocking antibodies at the concentrations used, TRAIL-sensitive RT4 bladder cancer cells were subjected to TRAIL as a positive control (Figure 6A, insert). As expected, TRAIL killed the cells and DR4/DR5 inactivation totally prevented TRAIL-induced apoptosis. To determine whether ciglitazone-triggered apoptosis is dependent on a variation of death receptor expression, Ca Ski cells were exposed to the drug and total protein extracts were analysed by western blotting for DR4 and DR5 expression. Ciglitazone (40 μM) strongly increased up to 2 to 5-fold DR4 and DR5 expression respectively (Figure 6B). As evidenced by flow cytometry with specific PE-conjugated antibodies directed against death receptors, the mean fluorescence intensity of DR4 and DR5 antigens was higher in ciglitazone-treated cells compared to control cells (Figure 6C). Thus, the drug raised also cell surface DR4 and, to a higher extent, DR5 expression.

### Ciglitazone upregulates soluble and membrane-bound TRAIL

We examined whether ciglitazone modifies TRAIL expression in Ca Ski cells. At a concentration of 40 μM ciglitazone, a 3.2-fold increase of TRAIL level was observed from total protein extracts analysed by western blotting (Figure 6D). As revealed by flow cytometry with a specific PE-conjugated TRAIL antibody, the mean fluorescence intensity of TRAIL antigen was higher in ciglitazone-treated cells compared to control cells indicating an increase of membrane-bound TRAIL (Figure 6E). In addition, a high increase of soluble TRAIL level was detected by ELISA assay in cell conditioned media upon drug treatment (Figure 6F). These data indicate that ciglitazone raised both soluble and cell surface TRAIL expression.

### Ciglitazone sensitizes TRAIL-resistant Ca Ski cells to TRAIL-induced apoptosis

**Figure 7:**
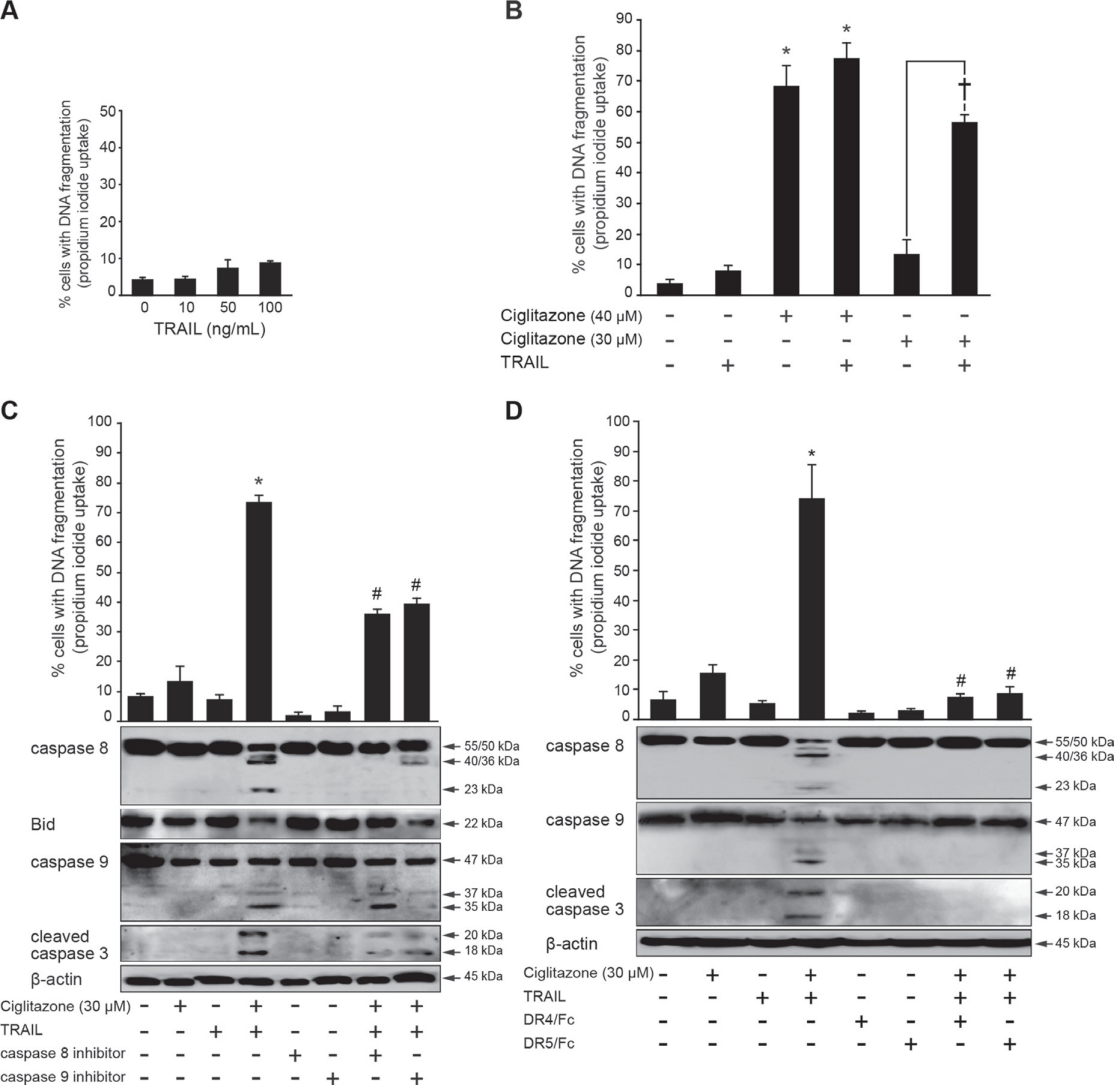
Ciglitazone lets TRAIL-resistant Ca Ski cells to respond to TRAIL (**A**) Cells were treated with TRAIL at the indicated concentrations. The percentage of cells showing hypodiploid DNA content (sub-G1 peak) was evaluated by flow cytometry analysis. (**B**) Ca Ski cells were treated or not with ciglitazone (30 or 40 μM) or human recombinant TRAIL (50 ng/ml) for 12 h or cotreated with ciglitazone and TRAIL. In this case, the duration of the treatment is 24 h (12 h with ciglitazone plus 12 h with TRAIL). The percentage of cells showing hypodiploid DNA content (sub-G1 peak) was evaluated by flow cytometry analysis. (**C**) Cells were pre-incubated for 1 h with 50 μM caspase 8 (Z-IETD-FMK) or 9 (Z-LEHD-FMK) specific inhibitor before indicated treatment; *top*, the percentage of cells showing hypodiploid DNA content (sub-G1 peak) was evaluated by flow cytometry analysis; *bottom*, cells were treated as indicated. Whole cell lysates were prepared and total protein extracts were subjected to immunoblotting for detection of caspase 8, 9, 3 and Bid processing. (**D**) Cells were pre-incubated with or without monoclonal antibodies blocking DR4 and DR5 receptors for 1 h and stimulated as indicated; *top*, the percentage of cells showing hypodiploid DNA content (sub-G1 peak) was evaluated by flow cytometry analysis; *bottom*, caspase 8, 9, and 3 cleavage was assayed by western blotting analysis. Data are means ± SEM of three independent experiments performed in triplicates. ^*^P < 0.05 compared to untreated cells. ^†^*P* < 0.05 compared to 30 μM ciglitazone-treated cells. ^#^*P* < 0.05 compared to ciglitazone and TRAIL cotreated cells. β-actin was used as an internal loading control.

Beforehand, we assessed the responsiveness of Ca Ski cells to increasing concentrations of TRAIL ranging from 10 to 100 ng/ml. The flow cytometric analysis of the sub-G1 population displayed that cells were highly refractory to TRAIL (Figure 7A). In accordance with our results previously mentionned, we proposed that ciglitazone triggers Ca Ski cell death by restoring their responsiveness to TRAIL. To confirm this assumption, cells were stimulated by ciglitazone or TRAIL alone or in association. In each case, two doses of ciglitazone were tested. A concentration of 40 μM induced 70% of cell death compared to 15% with a concentration of 30 μM. The combination of 40 μM ciglitazone and 50 ng/ml TRAIL did not increase apoptotic cell death compared to ciglitazone treatment alone. On the contrary, 30 μM ciglitazone acted synergistically with TRAIL to promote apoptosis (Figure 7B, 7C upper panel). In addition, the TRAIL-sensitizing effect of ciglitazone resulted in caspase 8, 9 and 3 cleavage and Bid truncation (Figure 7C, lower panel). The combination of caspase 8 (Z-IETD-FMK) or caspase 9 (Z-LEHD-FMK) specific inhibitors with ciglitazone and TRAIL decreased the sensitizing action of the TZD on TRAIL-induced apoptotic cell death as revealed by a significant decrease of cells with fragmented DNA (Figure 7C, upper panel). As predicted, the processing of caspase 8, 9 and 3 was inhibited and the Bid proform was still decreased in the presence of the caspase 9 inhibitor and was restored with the caspase 8 inhibitor (Figure 7C, lower panel). To validate the activation of death receptors in the sensitization of Ca Ski cells to TRAIL by ciglitazone, DR4 and DR5 activity was prevented by pre-incubation of cells with blocking anti-DR4 or anti-DR5 before the stimulation. The inactivation of both receptors upon TRAIL and ciglitazone cotreatment efficiently inhibited cell death as revealed by the decrease of cells with fragmented DNA and the inhibition of the caspase cascade establishing that the apoptotic process involved specific interactions between TRAIL and DR4/DR5 (Figure 7D).

### Ciglitazone inhibits c-FLIP expression

**Figure 8:**
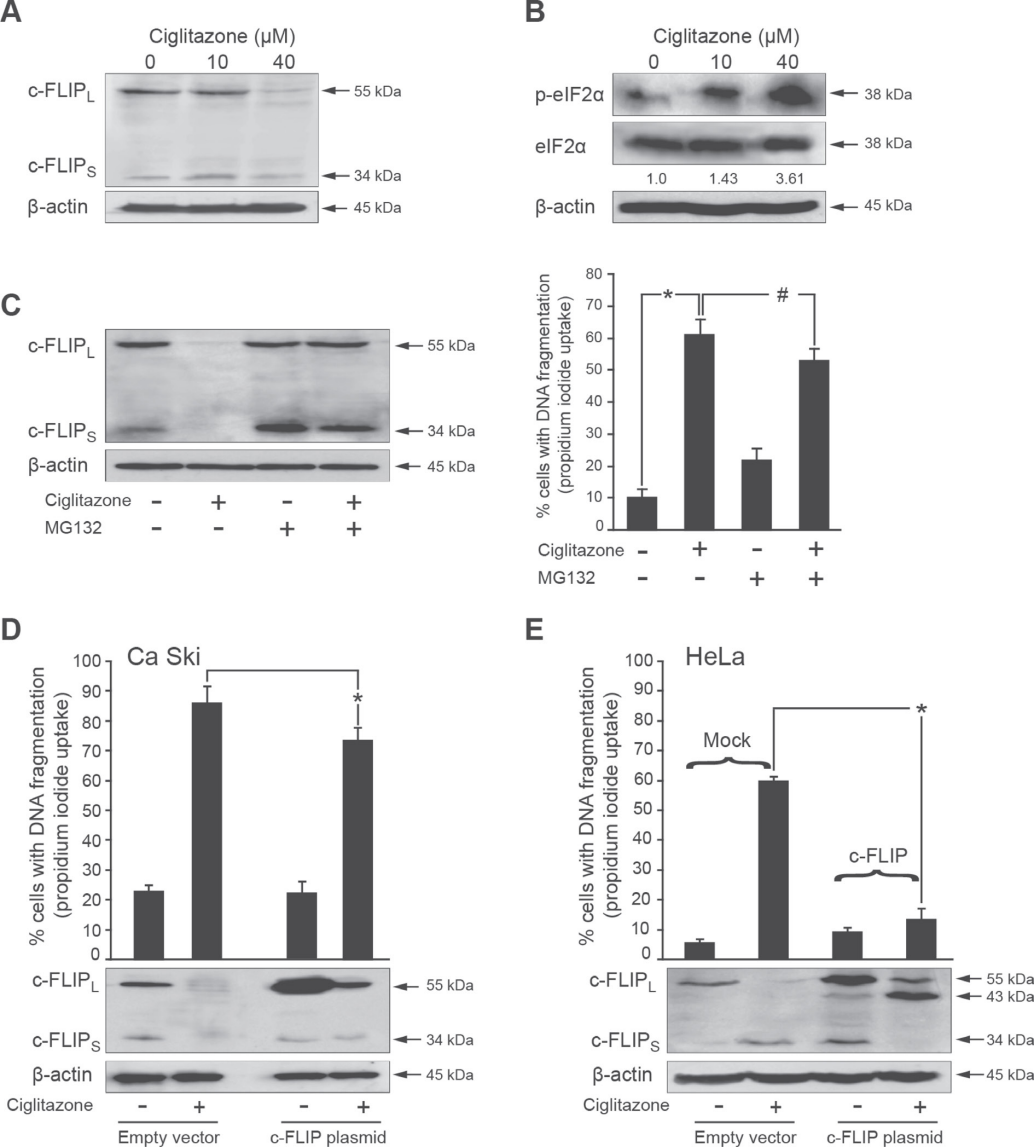
Down-regulation of c-FLIP is involved in ciglitazone-mediated apoptosis (**A**) Ca Ski cells were treated with ciglitazone at the indicated concentrations. Whole cell lysates were prepared and total protein extracts were subjected to immunoblotting for detection of c-FLIP. (**B**) Ciglitazone increases eIF2α phosphorylation. Whole cell lysates were prepared and total protein extracts were subjected to immunoblotting with a specific polyclonal antibody raised against phosphorylated eIF2α at serine 51. Levels of total eIF2α were assayed by using an antibody that recognizes both phosphorylated and non-phosphorylated versions of the translation initiation factor. Values of densitometric analyses are indicated. (**C**) Cells were pretreated with 25 μM proteasome inhibitor MG132 for 30 min and stimulated with 40 μM ciglitazone for 12 h. Cell lysates were prepared and subjected to western blotting analysis for detection of c-FLIP. Data are means ± SEM of two independent experiments performed in triplicates. ^*^P < 0.05 compared to untreated cells; ^#^*P* < 0.05 compared to ciglitazone-treated cells. (**D**) Ca Ski cells were transfected with c-FLIP (VSV tagged) construct or empty vector and stimulated for 12 h with 40 μM ciglitazone; *top*, the percentage of cells showing hypodiploid DNA content (sub-G1 peak) was evaluated by flow cytometry analysis. ^*^P < 0.05 compared to ciglitazone-treated cells. *Bottom*, cell lysates were prepared and subjected to western blotting analysis for detection of c-FLIP. (**E)** Mock HeLa cells and stable c-FLIP transfected HeLa cells were treated with 40 μM ciglitazone for 12 h; *top*, the percentage of cells showing hypodiploid DNA content (sub-G1 peak) was evaluated by flow cytometry analysis; *bottom*, cell lysates were prepared and subjected to western blotting analysis for detection of c-FLIP. β-actin was used as an internal loading control. Data are means ± SEM of two independent experiments performed in triplicates. ^*^P < 0.05 compared to ciglitazone-treated mock HeLa cells.

We also analysed the action of ciglitazone on the expression of c-FLIP known to be a master negative regulator of the extrinsic apoptotic pathway. As presented in Figure 8A, ciglitazone drastically reduced c-FLIP_L_ and c-FLIP_S_ protein levels at a concentration of 40 μM for which it promoted apoptosis. These results indicate that downregulation of c-FLIP is associated with ciglitazone-induced apoptosis.

### Reduced expression of c-FLIP is associated with an increase of eIF2α phosphorylation

The phosphorylation of the alpha subunit of the eukaryotic translation initiation factor 2 (eIF2) at Ser51 results in the global inhibition of protein synthesis in response to stress conditions [19]. In the experiment presented in Figure 8B, eIF2α phosphorylation was analysed by western blotting using a specific antibody for the phosphorylated form of eIF2α at Ser51. In Ca Ski cells, eIF2α phosphorylated form was 3.6-fold raised by 40 μM ciglitazone. These results suggest that ciglitazone induced an eIF2α phosphorylation increase which could contribute to protein synthesis inhibition of c-FLIP.

### Ciglitazone induces proteasome-dependent degradation of c-FLIP

C-FLIP is known to be degraded by a proteasome-mediated pathway [20, 21] which plays a central role in the regulation of apoptosis [22]. To find out whether the inhibition of c-FLIP by ciglitazone is mediated through this process, we examined the impact of the proteasome inhibitor MG132. In the presence of MG132, the levels of c-FLIP reduced by ciglitazone were restored (Figure 8C, left panel) indicating that ciglitazone-induced downregulation of this apoptosis inhibitor is proteasome dependent. In addition, we noticed that the number of dead Ca Ski cells was decreased when MG132 was associated with ciglitazone (Figure 8C, right panel).

### Enforced expression of ectopic c-FLIP decreases ciglitazone-mediated Ca Ski cell apoptosis

To test the importance of c-FLIP in ciglitazone-induced apoptosis, the effect of c-FLIP overexpression was examined either by the transient transfection of a c-FLIP plasmid or the use of stable c-FLIP transfected HeLa cells. As presented in Figure 8D (upper panel), the effect of ciglitazone on Ca Ski cell death was significantly diminished from 85% to 70% in Ca Ski cells overexpressing ectopic c-FLIP. As expected, in Ca Ski cells transfected with the empty vector, c-FLIP expression was abolished upon ciglitazone treatment. In Ca Ski cells overexpressing c-FLIP, ciglitazone partially decreased c-FLIP level compared to control cells (Figure 8D, lower panel). This could explain why TZD-mediated apoptosis was not totally counteracted when c-FLIP was overexpressed. In the Figure 8E, we used an other model of cervical cancer cells that was kindly provided in which c-FLIP was stably transfected. Ciglitazone induced mock HeLa cell apoptosis and this was associated with a dramatic decrease of c-FLIP_L_ protein expression (Figure 8E, lower panel). On the contrary, in stable c-FLIP transfected HeLa cells, the effect of ciglitazone on cell death was abolished. Interestingly, we detected the production of p43-FLIP known to result from the caspase 8-mediated processing of the N-terminal fragment of c-FLIP_L_. These results indicate that enforced expression of c-FLIP confers resistance to ciglitazone-induced apoptosis in stable c-FLIP transfected HeLa cells.

## DISCUSSION

A major hindrance in efficacious cancer therapy is the pre-existing or acquired resistance to chemotherapeutic drugs leading to the therapy failure and the disease recurrence. Therefore, the development of novel antitumour agents is required to overcome chemo-resistant cancer cells.

Exposure to PPARγ agonists such as TZD has been shown to trigger apoptosis in a variety of cancers using *in vitro* and animal models through PPARγ-dependent or -independent mechanisms [23]. Especially, several studies reported the part played by ciglitazone used as a single agent [24–28] or in combination with existing chemotherapeutic agents [29–32] in induction of tumour cell apoptosis.

In this study, we explored the therapeutic possibility of ciglitazone as an anticancer agent in cervical cancer both *in vitro* and *in vivo*. Very few studies reported the effects of TZD on cervical cancer cells. The anticancer action of troglitazone in HeLa cells was associated with apoptosis induction through the inhibition of p53 ubiquitination [33]. The few published data reporting the effect of ciglitazone in cervical cancer cells (C-33 A, C-4II, HeLa) showed either an inhibition of the proliferation or an apoptotic death in a PPARγ-independent manner [34, 35]. Here, we presented evidence that this drug triggered apoptotic cell death in a dose- and time-dependent manner. In particular, we revealed in HPV16-infected Ca Ski cells the underlying mechanisms involved in ciglitazone-induced apoptosis. As evidenced by the caspase 8, 9 and 3 activation as well as the cleavage of Bid, both extrinsic and intrinsic apoptotic signalling pathways were likely implicated. Interestingly, we showed for the first time in Ca Ski cells that the PPARγ agonist-mediated cell death was associated with soluble and membrane-bound TRAIL upregulation whereas these cells were refractory to TRAIL.

TRAIL overexpression-mediated apoptosis has been reported in different cancer models. For instance, TRAIL increase was induced by live *Lactobacillus casei* in colon carcinoma cells [36], IL-9 in HTB-72 melanoma cells [37], or IPS-1 (IFN-β promoter stimulator-1) in human IMR-32 neuroblastoma cells [38]. TRAIL is considered as an attractive anticancer molecule as it specifically induces tumour cell apoptosis while sparing the normal cells [5]. However, a large number of cancers including cervical cancer develop a resistance towards TRAIL potentially limiting its therapeutic utility. Several crucial cellular processes including defective protein synthesis, protein misfolding, ubiquitin regulated death receptor expression, metabolic pathways, epigenetic deregulation and metastasis could contribute to TRAIL resistance [39]. DR5 and c-FLIP have been reported to be molecular targets leading various tumour cells to resist to TRAIL-induced apoptosis. The combination of TRAIL with novel compounds used to restore TRAIL sensitivity represents an attractive clinical option. TZD have notably the ability to sensitize tumour cells to TRAIL-induced cell death but the underlying mechanisms remain poorly understood. Nevertheless, they could involve the induction of p21^waf1/cip1^ [40], the inhibition of G1/S cell cycle progression [41], the downregulation of c-FLIP and survivin [24, 42, 43], the ROS-mediated upregulation of DR5 [44], the phosphorylation of Bad [45], the decrease of β-catenin expression [46], the overexpression of DR5 associated with a decrease of XIAP [47], the decreased expression of cyclin D3. But until now, no data were available in cervical cancer cells.

In this study, we showed that the up-regulation of both agonistic receptors DR4/DR5 could contribute to ciglitazone sensitizing action on TRAIL-resistant Ca Ski cell apoptosis. Several mechanisms for PPARγ agonist-mediated upregulation of DR5 have been already reported. Rosiglitazone increases DR5 protein level in a dose and time-dependent manner through ROS generation [44]. The 15-deoxy-Δ-^12,14^-prostaglandin J2, known as the endogenous ligand of PPARγ, induces cell death through DR5 mRNA stabilization [48]. The 3′-untranslated region of the human *DR5* gene contains AU-rich elements, at least two overlapping copies of the UUAUUUAUU monomer. Thus, 15-deoxy-Δ-^12,14^-prostaglandin J2 might affect AU-rich elements at the post-transcriptional level. Otherwise, the *DR5* gene was reported as a p53-regulated gene [49–51]. A recent study reported that the orally available tyrosine kinase inhibitor BAY61-3606 sensitized colon cancer cells to TRAIL-induced apoptosis through the upregulation of DR4 in a p53-dependent mechanism [52]. In the present work, we showed that p53 was increased upon ciglitazone exposure and was transcriptionally active since the p53-target p21 was upregulated. Thus, we can speculate that p53 could be involved in DR4/DR5 upregulation. Further experiments are needed to clarify the ciglitazone-mediated molecular mechanisms implicated in death receptor transcriptional regulation.

C-FLIP is a catalytically inactive caspase 8/caspase 10 homologue. It is expressed as three major isoforms in humans: c-FLIP_L_, c-FLIP_S_ and c-FLIP_R_. All c-FLIP isoforms contain two DED that are structurally similar to the N-terminal part of procaspase-8. C-FLIP_L_ contains additional caspase-like domains (p20 and p12) that are catalytically inactive. C-FLIP_S_ additionally has an isoform specific C-terminal tail of 19 amino acids. Furthermore, two N-terminal cleavage products of 43 and 22 kDa (named p43-FLIP and p22-FLIP) result from the caspase 8-mediated cleavage of c-FLIP_L_ at specific caspase cleavage sites. Both c-FLIP_S_ and c-FLIP_L_ are recruited to the DISC through DED interactions and block caspase 8 processing and thus death receptor-induced apoptosis [21]. C-FLIP has been reported to be overexpressed in several types of cancer. In particular, its overexpression is related to cervical cancer progression [53]. In addition, high expression of c-FLIP correlates with TRAIL resistance in cancer cells, and c-FLIP-mediated TRAIL resistance can be overcome by combining with c-FLIP targeting chemotherapeutics or c-FLIP siRNA. Targeting c-FLIP is thus a relevant therapeutic option to treat cancer and TRAIL or chemotherapeutic drug resistance of tumour cells [54]. And precisely, we demonstrated that ciglitazone treatment reduced both c-FLIP_L_ and c-FLIP_S_ levels. Several mechanisms underlie TZD-mediated downregulation of anti-apoptotic regulators of the apoptotic signalling cascade namely, ubiquitination and proteasome-dependent degradation with or without transcriptional events [42, 55, 56]. Moreover, TZD have been reported to inhibit translation initiation by phosphorylating eIF2α [57, 58]. In particular, ciglitazone has been shown to cause calcium release from the endoplasmic reticulum leading to the activation of several protein kinases such as PERK and PKR that phosphorylate eIF2α [57]. In addition, it has been shown that inhibition of eIF2α dephosphorylation could lead to TRAIL-induced apoptosis in hepatoma cells [59]. In our study, we first found that eIF2α was phosphorylated by ciglitazone treatment in Ca Ski cells suggesting that this drug promoted inhibition of the translation initiation of c-FLIP. Second, the downregulation of c-FLIP was mediated by a proteasomal degradation. However, transient forced expression of c-FLIP was not sufficient to completely prevent ciglitazone-induced apoptosis in Ca Ski cells suggesting the involvement of an other anti-apoptotic regulator that has to be determined. On the contrary, stably transfected HeLa cells with c-FLIP were resistant to ciglitazone-triggered cell death.

The maintenance of the transformed phenotype of cervical cancer cells depends upon the constant expression of both viral E6 and E7 oncoproteins [60]. In particular, E6 promotes p53 proteasomal degradation [61]. Importantly, E6 can disrupt apoptotic signalling pathways triggered by TNF, TRAIL and FasL. This inhibiting capability is mediated by E6 binding to and degradation of both FADD adapter protein and the effector caspase 8 [62, 63]. Different strategies have been performed to block E6 expression or activity in HPV-harbouring cancer cells such as siRNA [64], antisense RNA [65] or anticancer drugs [66]. We presented the first evidence that ciglitazone led to the downregulation of E6 at both the mRNA and protein levels. The molecular mechanisms involved have to be elucidated. In human cervical cancers, the methylation of HPV promoter could favour the expression of E6. PPARγ agonists such as ciglitazone and 15-deoxy-Δ-^12,14^-prostaglandin J2 elicit Foxp3 DNA demethylation in human induced regulatory T cells through the downregulation of DNMT [67]. We can speculate that ciglitazone could decrease *e6* oncogene expression through the promoter demethylation. Further investigations are needed to clarify this point. It is well known that binding of E6 protein to E6-associated protein (E6AP) is necessary for E6-mediated acceleration of p53 degradation [68]. We highlighted that ciglitazone decreased E6 level and we indeed demonstrated an increase in the level of p53 transcriptionally active proteins in the cells treated with ciglitazone. As previously discussed, DR5 is transcriptionally regulated by p53. We could hypothesize that ciglitazone-mediated decrease of E6 could lead to the increased expression of DR4/DR5 through p53 expression restoration. Complementary experiments are required to clear up this point.

**Figure 9:**
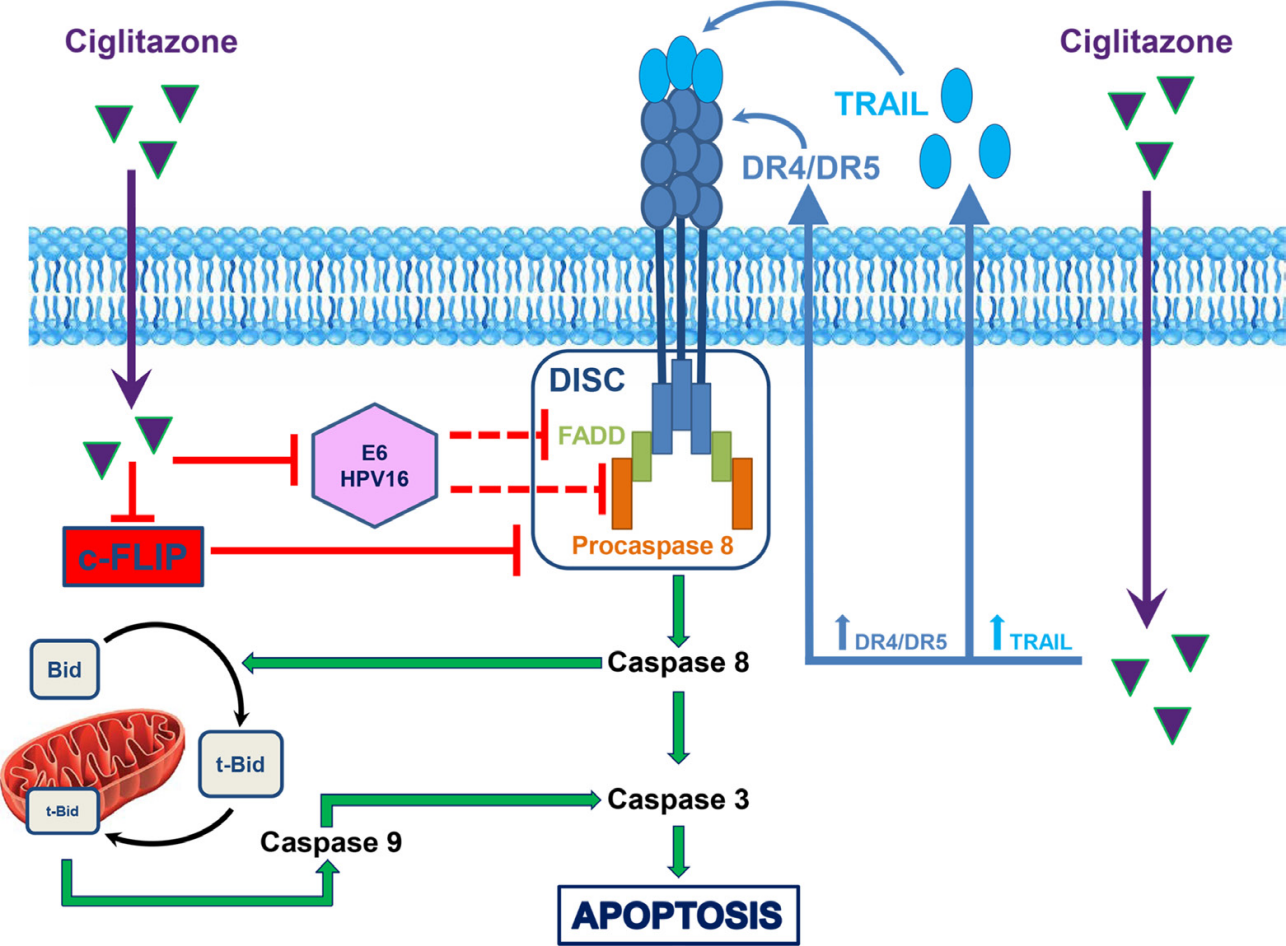
Schematic summary of ciglitazone action on TRAIL-resistant Ca Ski cervical cancer cells After entry of ciglitazone into the cell (purple arrow), it increases DR4/DR5 as well as TRAIL expression (blue arrow) leading to the activation of the caspase cascade and thus to apoptosis (green arrow). Extrinsic and intrinsic apoptotic pathways are interconnected by the caspase 8-mediated cleavage of Bid (tBid, truncated Bid) induced by ciglitazone. The thiazolidinedione decreases HPV16 E6 viral oncoprotein (red arrow) reported to block TRAIL pathway (refs. 62, 63, red dotted arrow). Ciglitazone sensitizes tumour cells to TRAIL-dependent cell death through the downregulation of the caspase 8 inhibitor c-FLIP (red arrow).

In summary, this work has established the pathway for the antitumour action of ciglitazone in cervical cancer cells (Figure 9). We demonstrated for the first time that the drug is able to delay cervical cancer development in xenografted mice. According to our study, it is required to elucidate the PPARγ-independent mechanisms of ciglitazone effect prior to clinical exploration. Nowadays, no chemical molecule targeting E6 is available for clinical use. In this context, ciglitazone blocking action on E6 could be a useful therapeutic option for HPV-associated malignancies. The role of E6 as a primary target for the different proapoptotic effects of ciglitazone warrants further investigations. More globally, this antidiabetic drug used as a single treatment or in combination with TRAIL may be a potential chemotherapeutic strategy for cancer in general since it targets several common apoptotic mediators (DR4/DR5, TRAIL, c-FLIP).

## MATERIALS AND METHODS

### Chemicals and reagents

Thiazolidinediones (rosiglitazone, pioglitazone, ciglitazone) and the PPARγ antagonist GW9662 were provided from Cayman Chemical (Ann Arbor, MI, USA). Soluble recombinant TRAIL, proteasome inhibitor MG132, caspase 9 specific inhibitor (Z-LEHD-FMK) and propidium iodide (PI) were purchased from Sigma (Saint Quentin Fallavier, France). Caspase 8 inhibitor (Z-IETD-FMK) was from Bachem (Heidelberg, Germany). Anti-DR4 and DR5 blocking antibodies were from Alexis Biochemicals (Lausanne, Switzerland).

### Cell culture

The human cervical carcinoma cell lines HeLa, Ca Ski, C-33 A and the human bladder cancer cell lines RT4 and T24 were obtained from ATCC (Rockville, MD, USA). The stable cell line HeLa-c-FLIP was a kind gift from Dr. O. Micheau (Bourgogne Franche-Comté University, Dijon, France). Cells were maintained in DMEM (HeLa, Ca Ski, stable HeLa-c-FLIP), EMEM (C-33 A) or Mc COY's 5A medium (RT4, T24) supplemented with 10% fetal calf serum (FCS) (Invitrogen, Cergy Pontoise, France), 1% antibiotic antimycotic mixture (10 mg/ml streptomycin, 10 000 U/ml penicillin, 25 μg/ml amphotericin B), 2 mM glutamine and 15 mM Hepes (Sigma) at 37°C in a humidified 5% CO_2_, 95% O_2_ air incubator. Cells were tested for the absence of mycoplasma before the beginning of experiments.

### Cell death analysis by flow cytometry

Cells were seeded in triplicates (6.10^3^ cells/cm^2^) in 12-well plates and incubated in culture medium supplemented with 5% decomplemented FBS. After 24 h, they were exposed to the indicated concentrations of thiazolidinediones for 24 h or to 40 μM ciglitazone for 4, 12, 16, 18, 20, 24 h in serum-free culture medium. DNA fragmentation was measured by PI staining and fluorescence-activated cell sorting (FACS) analysis (FC 500 Beckman Coulter) as previously described [24]. Twenty thousand events were analysed per sample and apoptosis was determined by the Sub-G1 DNA content with CXP software (Beckman Coulter).

### Western blot analysis

After treatment with TZD, cells were washed with cold PBS 1X and scraped in RIPA lysis buffer (50 mM Tris-HCl pH 7.4, 150 mM NaCl, 1 mM EDTA, 1% Nonidet P40, 0.5% sodium desoxycholate) supplemented with protease inhibitors (Roche, Meylan, France). Then, whole cell lysates were sonicated and centrifuged at 10,000 rpm for 10 min at 4°C. Protein concentration was estimated using the Bio-Rad protein assay (Bio-Rad, Marnes-la-Coquette, France). Total protein extracts (15 μg) were solved in Laemmli buffer (Bio-Rad) and separated by a 7.5, 12 or 15% SDS-PAGE. Proteins were transferred onto PVDF membranes (GE Healthcare, Buckinghamshire, UK) and non specific binding was blocked in TBS-Tween 20 buffer (0.5 mM Tris-HCl, 45 mM NaCl, 0.05% Tween 20, pH 7.4) containing 5% nonfat milk. Membranes were incubated with the following appropriate primary antibodies: anti-caspase 8 (clone 3-1-9, 1:1000), anti-p21^Cip1/Waf1^ (clone 6B6, 1:500), anti-p53 (clone DO-1, 1:500), and anti-PARP (clone 4C10-5, 1:1000) that were obtained from BD Pharmingen (BD Biosciences, Le Pont de Claix, France). Anti-caspase 9 (#9502, 1:1000), anti-cleaved caspase 3 (#9661, 1:1000), anti-Bcl-2 (#2876, 1:1000), anti-Bax (#2772, 1:1000), anti-Bid (#2002, 1:500), anti-eIF2α (#9722, 1:500), and anti-phospho eIF2α (#3597, 1:500) were from Cell Signalling (Ozyme, St Quentin en Yvelines, France). Mouse monoclonal anti-c-FLIP (clone NF6, 1:500) was from Alexis Biochemicals. Rabbit polyclonal anti-DR4 (#AB16955, 1:1000), anti-DR5 (#AB16942, 1:1000) and anti-TRAIL (#AB16957, 1:500) were purchased from Merck Millipore (Molsheim, France). Anti-HPV16 E6 (clone 3F8, 1:1000) was from Euromedex (Souffelweyersheim, France). Anti-PPARγ (clone E8, 1:500) was from Santa Cruz (CliniSciences, Nanterre, France). Protein blots were probed with anti-β-actin (clone AC-15, 1:40,000, Sigma) as controls for protein loading. Bound primary antibodies were detected using HRP-conjugated secondary antibodies: anti-rabbit IgG (1:5000 or 1:10,000) or anti-mouse IgG (1:10,000) provided from BD Pharmingen. Proteins were visualized by using enhanced chemiluminescence detection method (GE Healthcare) followed by film exposure (Hyperfilm ECL, GE Healthcare). Densitometric analysis was performed with the software Image J.

### Cell transfection and dual luciferase reporter assay

The pCG-E6 construct encoding HPV E6 oncoprotein was a gift from Dr. F. Thierry (Singapore). The c-FLIP (VSV tagged) plasmid was kindly gifted by Dr. O. Micheau. Ca Ski or C-33 A cells were plated in 24-well plates at a density of 100,000 cells per well in DMEM or EMEM supplemented with 5% decomplemented FBS and were grown to 60% confluence. They were transfected with 1 μg plasmid DNA and 2 μl of jetPEI^TM^ transfection reagent (Eurogentec, Angers, France) as specified by the manufacturer's recommendations for 24 h. At 24 h post-transfection, cells were stimulated or not by ciglitazone for 12 h and then harvested for flow cytometry and protein analyses. For luciferase reporter assays, HeLa, Ca Ski and C-33 A cells were cotransfected with the reporter plasmid Cyp2XPal-luc (a kind gift from Pr. L. Michalik, Lausanne, Switzerland) containing 2 copies of a PPRE sequence located upstream from the firefly luciferase gene, and the pRL-CMV plasmid containing Renilla luciferase (Promega, Charbonnières-les-Bains, France) used as an indicator of transfection efficiency. At 24 h after transfection, cells were treated or not with ciglitazone in serum-free culture medium for additional 24 h and then were harvested using Passive Lysis Buffer (Promega). Firefly and Renilla luciferase activities were measured subsequently using Dual-Luciferase Reporter Assay System (Promega) according to the manufacturer's instructions.

### SiRNA transfection

The non-targeting siRNA and PPARγ siRNA (100 nM) duplexes were purchased from Tebu Bio (Le Perray-en-Yvelines, France) and Eurogentec (Seraing, Belgium) respectively. Ca Ski cells were seeded in 24-well plates and transfected at 60% confluence in serum-free conditions using Lipofectamine^TM^ 2000 transfection reagent according to the manufacturer's instructions (Invitrogen). After 24 h of transfection, siRNA were removed and cells were incubated or not with 40 μM ciglitazone for additional 12 h and then were harvested for protein expression.

### RNA extraction, reverse transcription, and quantitative real-time PCR

Total RNA were isolated from Ca Ski and C-33 A cells using TRIzol^®^ reagent (Invitrogen) and cDNA were synthesized with the Reverse Transcription System^®^ kit (Promega) according to the manufacturer's recommendations. Quantitative PCR was performed using specific primers and probe for *HPV16 e6* (forward 5′-TTTTATGCACCAAAAGAGAACTGC-3′ and reverse 5′-AGCTCTGTGCATAACTGTGGTAACTT-3′; probe: 5′-BHQ1-CAGGAGCGACCCAGAAAGTTACCACAG TT-FAM-3′). The housekeeping gene β2-microglobulin (β2-M) was used as a reference gene for normalization with the following primers and probe: forward 5′-GATGAGTATGCCTGCCGTGTG-3′ and reverse 5′-CA ATCCAAATGCGGCATCT-3′; probe: 5′-FAM-CCTCCAT GATGCTGCTTACATGTCTCGATCCC-TAMRA-3′. Quantification of the mRNA was performed using the 7500 Real Time PCR System (Applied Biosystems, Courtaboeuf, France) with the TaqMan technology in a final volume of 25 μl containing 12.5 μl of TaqMan Gene Expression PCR Master Mix (Applied Biosystems), 5 μl of cDNA diluted 1:20, 50 nM of TaqMan probe (Eurogentec, Seraing, Belgium), and 0.5 μM of each primer (Eurogentec) for both *HPV16 e6* and *β2-M* genes. The Taq polymerase was activated at 50°C for 2 min, followed by a denaturation step at 95°C for 10 min. Then, PCR mixtures were subjected to 40 cycles of amplification. The following PCR cycle settings were used: denaturation at 95°C for 15 sec, hybridization/elongation at 60°C for 1 min. Each reaction was run in triplicates with three independent triplicates. We calculated the relative mRNA expression level using a comparative CT method [69]. *Β*2-M was used for the reference gene. The normalized relative expression level of a target gene in an individual sample was calculated using the following formula : (E_target_)^ΔCT target (calibrator-sample)^/(E_reference_)^ΔCT reference (calibrator-sample)^ in which the real-time PCR efficiency of the target gene transcript is donated by E_target_ and that of the reference gene transcript, by E_reference_. Thus, the relative mRNA expression level of a gene is a unit less number relative to that of the calibrator sample.

### Tumour xenograft model

Five to eight-week-old female *nu/nu* nude mice were purchased from Charles River (L’Arbresle, France) and maintained according to European Union guidelines for use of laboratory animals. *In vivo* experiments were performed in compliance with the French guidelines for experimental animal studies. Exponentially growing Ca Ski cells were subcutaneously injected into the right flank of female nude mice as follows: 5 × 10^6^ cells in 50 μl PBS with 25% decomplemented FCS. Animals were examined twice a week for tumour development. Tumour size was monitored twice a week by measuring length and width with a calliper. Tumour volume was estimated using the following formula: volume = width^2^ × length × ½. When tumour size reached 40 mm^3^ in volume, mice were randomly divided into control and treated groups containing ten mice each. Intraperitoneal injections of ciglitazone were weekly administered at a dose of 15 mg/kg during three weeks. Control animals received only saline vehicle following an identical schedule. Mice were sacrificed one week after the third injection and tumours were removed for analyses.

### Immunohistochemistry

Vehicle- and ciglitazone-treated tumours were collected, fixed in 4% formalin and paraffin-embedded for immunohistochemical analyses as previously described [24]. The biotinylated primary antibody anti-Ki-67 (DakoCytomation, MIB-1, 1:150) was used for evaluation of proliferating cells. Activated caspase 3 was detected with Apoptosis marker: SignalStain cleaved Caspase 3 (Asp175) IHC Detection kit (Cell Signalling). Slides were then treated with alkaline phosphatase-conjugated avidin. Finally, alkaline phosphatase activity was revealed by NBT-BCIP. Sections were counterstained with Harris’ hematoxylin, dehydrated through alcohol and mounted using a standard procedure. Negative controls were obtained by omitting the first antibody. Slides were then examined and pictures were taken using Zeiss Axioskop 40 photomicroscope.

### Detection of DR4, DR5 and TRAIL cell surface expression

Ca Ski cells were treated in serum-free medium with ciglitazone alone or in combination with TRAIL as indicated. They were detached with 5 mM EDTA and centrifuged at 1 200 rpm for 10 min. Cells (10^6^) were resuspended in 50 μl of PBS and stained with 10 μl of phycoerythrin (PE)-conjugated mouse monoclonal anti-human DR4 (clone B-N36), DR5 (clone B-K29) or TRAIL (clone B-S23) provided by Diaclone (Besançon, France), for 30 min at 4°C in the dark. Cells were then washed twice in 1% BSA-PBS and resuspended in 50 μl of PBS. Flow cytometry analyses (FC 500 Beckman Coulter) were performed at a 580 nm wavelength. PE-conjugated mouse IgG1 were used as an isotype control.

### Determination of TRAIL production

Ca Ski cells (3.10^5^) were seeded in triplicates in 6-well plates and cultured in DMEM with 5% FCS for 24 h. The following day, cells were stimulated in serum-free medium with 40 μM ciglitazone for 24 h. Supernatants were collected and TRAIL was quantified by enzyme-linked-immunosorbent assay (Diaclone).

### Statistical analysis

For *in vitro* experiments, two-tailed unpaired Student's *t*-test was used to determine the significant differences between groups. Data are expressed as mean ± SEM of three independent experiments or as specified for each figure. The *in vivo* therapeutic efficacy of ciglitazone was assessed by evaluating the tumour volume development over time with two-way ANOVA test. Differences between control and treated mice at each post-graft time were determined by Student's *t*-test. *P*-values < 0.05 were considered to be significant.

## Abbreviations

Apaf-1: Apoptotic peptidase activating factor 1
BCIP: 5-bromo-4-chloro-3-indolyl-phosphate
Bid: BH3 interacting-domain death agonist
c-FLIP: cellular FLICE-Like Inhibitory Protein
DED: death effector domain
DISC: death-inducing signalling complex
DNMT: DNA methyltransferase
DR: death receptor
eIF2: eukaryotic initiation factor 2
FADD: Fas-associated protein with death domain
HPV: Human papillomavirus
NBT: nitro blue tetrazolium
PARP: Poly(ADP Ribose) Polymerase
PERK: PKR-like endoplasmic reticulum kinase
PKR: double-stranded RNA-activated protein kinase
PPAR: Peroxisome Proliferator-Activated Receptor
PPRE: Peroxisome Proliferator Responsive Element
ROS: reactive oxygen species
RXR: Retinoid X receptor
TNF: tumour necrosis factor
TRAIL: TNF related apoptosis inducing ligand
TZD: thiazolidinedione.
